# Circulating tumor DNA molecular response as a complementary biomarker to RECIST in immune checkpoint inhibitor therapy: current evidence and future directions

**DOI:** 10.3389/fonc.2026.1931505

**Published:** 2026-09-17

**Authors:** Jian Zheng, Luyang Chi, Yong Guo

**Affiliations:** Zhejiang Provincial Hospital of Chinese Medicine, The First Affiliated Hospital of Zhejiang University of Traditional Chinese Medicine, Hangzhou, China

**Keywords:** circulating tumor DNA, immune checkpoint inhibitors, liquid biopsy, molecular response, pseudoprogression, RECIST, surrogate endpoint

The emergence of immune checkpoint inhibitors (ICIs) has transformed the treatment landscape of multiple solid tumors. However, conventional radiographic evaluation using Response Evaluation Criteria in Solid Tumors version 1.1 (RECIST v1.1) remains challenged by delayed response assessment and immune-related atypical response patterns, including pseudoprogression. Circulating tumor DNA (ctDNA) molecular response has emerged as a promising minimally invasive biomarker capable of capturing dynamic tumor evolution earlier than conventional imaging (1).

Recent prospective studies, including the BR.36 adaptive trial and large pan-cancer immunotherapy cohorts, have demonstrated significant associations between early ctDNA dynamics, radiographic response, and survival outcomes. Nevertheless, current evidence remains insufficient to establish ctDNA molecular response as a validated surrogate endpoint replacing RECIST. Important challenges include heterogeneous response definitions, variability in assay methodologies, tumor-specific differences in ctDNA shedding, and the absence of randomized trials demonstrating improved outcomes through ctDNA-guided treatment adaptation.

In this perspective, we critically evaluate the current evidence supporting ctDNA molecular response assessment during ICI therapy and propose a ctDNA-enhanced RECIST framework (**Figure 1**). Within this framework, ctDNA should be considered a complementary biomarker that may facilitate early efficacy assessment, improve interpretation of pseudoprogression, and support treatment optimization. Future prospective randomized studies are required to determine whether ctDNA-guided strategies can improve clinical outcomes and establish its role in precision immunotherapy management.

**Figure 1:**
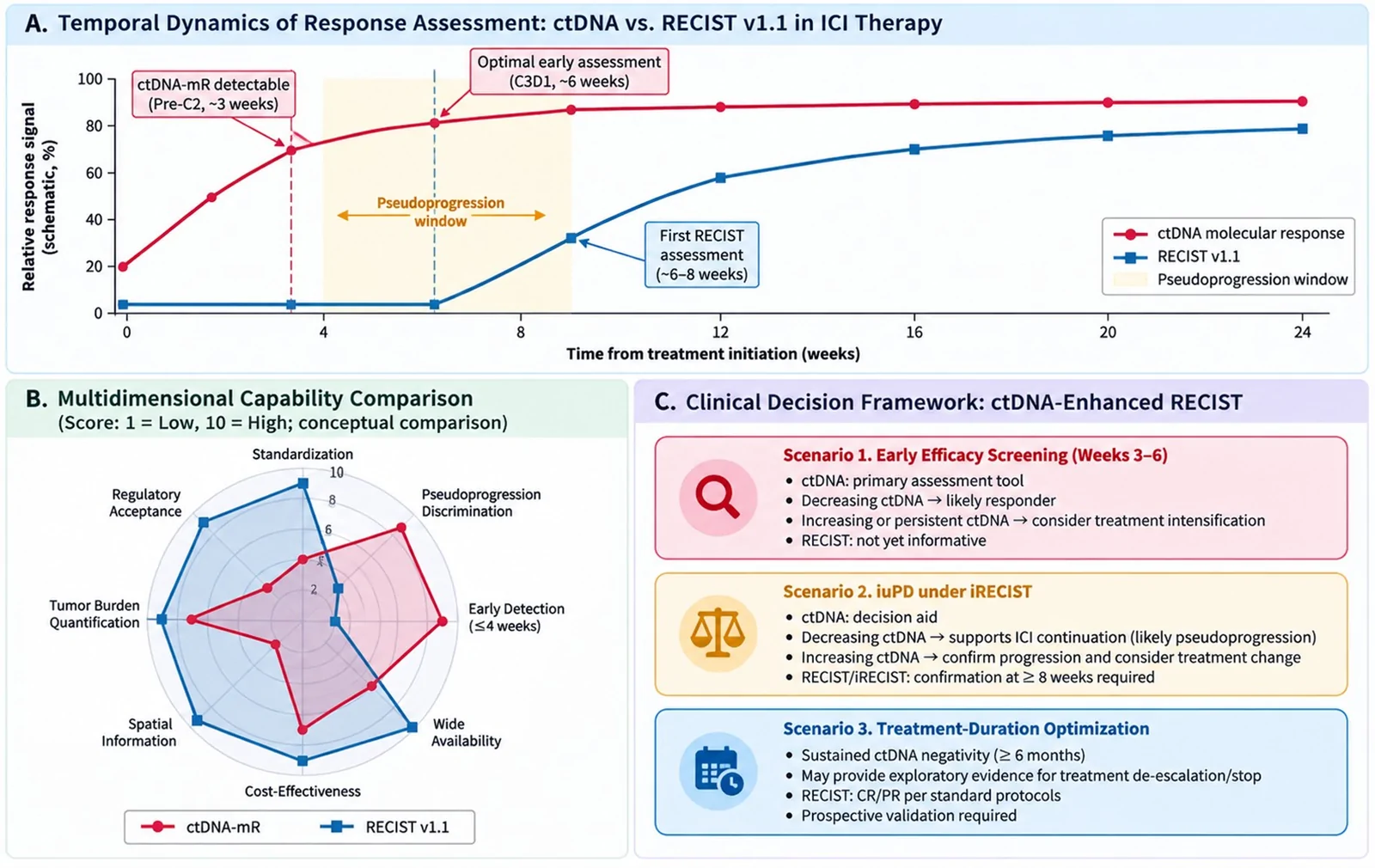
illustrates the complementary temporal and multidimensional profiles of ctDNA molecular response and RECIST v1.1. **(A)** demonstrates that ctDNA-mR becomes detectable as early as 3 weeks, whereas RECIST v1.1 remains non-informative until 6–8 weeks. **(B)** highlights the divergent strength profiles: ctDNA provides advantages in early molecular response assessment and may assist pseudoprogression interpretation, while RECIST maintains superiority in standardization, regulatory acceptance, and spatial resolution. Based on these complementary characteristics, we propose a ctDNA-enhanced RECIST framework **(C)**, in which ctDNA serves as an adjunct across three clinical scenarios: early efficacy screening, iuPD decision-making, and treatment duration optimization.

Given that circulating tumor DNA (ctDNA) has a biological half-life of approximately 30 minutes and reflects dynamic tumor burden changes, it has attracted considerable interest as an early, minimally invasive biomarker for monitoring treatment response. However, whether ctDNA molecular response can function as a validated surrogate endpoint remains uncertain and requires prospective clinical validation (2). The central question is whether ctDNA molecular response (ctDNA-mR), defined variably as ctDNA clearance or a predefined percentage reduction in variant allele frequency, can reliably augment RECIST-based assessments and improve early clinical decision-making in ICI trials.

Emerging prospective data support a meaningful concordance between ctDNA dynamics and radiographic response. The landmark phase 2 adaptive trial BR.36 (NCT04093167) enrolled 50 patients with treatment-naïve, PD-L1–positive (≥1%) advanced non-small cell lung cancer (NSCLC) receiving pembrolizumab monotherapy (3). Using a tumor-informed personalized assay, the investigators demonstrated that maximal mutant allele fraction clearance by cycle 3 day 1 (approximately 6 weeks) achieved a sensitivity of 82% (90% confidence interval [CI], 52–97%) and a specificity of 75% (90% CI, 56.5–88.5%) for RECIST-defined best overall response. Notably, the median time to ctDNA-mR was 2.1 months (90% CI, 1.5–2.6), preceding conventional restaging by several weeks. Patients who attained ctDNA-mR experienced significantly prolonged progression-free survival (PFS; 5.03 vs. 2.6 months) and overall survival (OS; not reached vs. 7.23 months), underscoring the prognostic fidelity of early molecular response. These findings informed the ongoing randomized second stage of BR.36, in which patients with molecular progression are allocated to treatment intensification versus pembrolizumab continuation. Complementing this, a large pan-cancer study from Vall d’Hebron Institute of Oncology (VHIO) analyzed 1,455 longitudinal plasma samples across 24 cancer types in 202 patients receiving diverse immunotherapy modalities, including ICI combinations, bispecific antibodies, and T-cell engagers (4). In this cohort, ctDNA-mR assessed as early as 3 weeks after treatment initiation correlated with improved PFS and OS. Patients achieving ctDNA molecular complete response (ctDNA-mCR; undetectable ctDNA at any timepoint) had a >3-fold increase in median PFS (6.4 vs. 1.9 months; P = 1.3 × 10^-4^) and a 5-year OS rate of 69%. Importantly, durable ctDNA-mCR maintained for ≥180 consecutive days (ctDNA-dmCR) was associated with 100% survival through the study period. Collectively, these findings indicate that ctDNA-mR represents a clinically informative early biomarker with strong prognostic associations, although its role as a validated surrogate endpoint remains to be established.

Beyond concordance, ctDNA offers unique advantages that transcend the inherent constraints of anatomical imaging. First, ctDNA enables the early identification of primary resistance. In the BR.36 trial, ctDNA-mR was detectable by 7 weeks, whereas RECIST responses were typically not evaluable until 12 weeks (3). In the VHIO cohort, only 7% of patients had clinically progressed by the pre-cycle 2 timepoint (approximately 3 weeks), yet ctDNA dynamics already stratified long-term outcomes, suggesting a window for early intervention in nonresponders (4). Second, ctDNA dynamics effectively resolve the pseudoprogression dilemma. Among 45 patients with immune unconfirmed progressive disease (iuPD) per iRECIST who continued immunotherapy, those with decreasing ctDNA immediately prior to iuPD demonstrated a 1-year OS rate of 80%, compared with 39% in patients with increasing ctDNA (4). This observation was corroborated in the validation cohort (67% vs. 16%). Furthermore, among patients with RECIST v1.1 progressive disease but concomitant decreasing ctDNA, continued immunotherapy was associated with significantly superior OS compared with patients harboring concordant radiographic and molecular progression. These findings align with the NADIM phase 2 trial in resectable stage IIIA NSCLC, where post-neoadjuvant ctDNA levels outperformed RECIST v1.1 assessments in predicting long-term survival (3-year OS, 81.9% in the intention-to-treat population) (5). Thus, ctDNA provides actionable biological information that imaging alone cannot capture, particularly in scenarios of treatment ambiguity.

Despite this promise, several critical barriers preclude the unqualified adoption of ctDNA-mR as a standalone surrogate endpoint for RECIST.

Current Limitations of Clinical Evidence Supporting ctDNA-Based Response Assessment

Although prospective studies have provided encouraging evidence regarding the prognostic value of ctDNA dynamics, several limitations currently restrict its implementation as a routine response assessment tool.

First, most available studies remain exploratory biomarker investigations or secondary analyses rather than prospective trials specifically designed to demonstrate clinical utility. Therefore, associations between ctDNA changes and survival outcomes should not be interpreted as evidence of treatment benefit resulting from ctDNA-guided decision-making.

Second, the generalizability of current findings remains uncertain. Studies such as BR.36 were conducted in selected patient populations, including PD-L1-positive NSCLC treated with pembrolizumab, whereas pan-cancer cohorts include heterogeneous tumor types, treatment regimens, and biological contexts. Whether ctDNA dynamics provide equivalent predictive value across different malignancies requires further investigation.

Third, differences in tumor biology, metastatic distribution, and baseline tumor shedding may influence ctDNA detectability. Therefore, absence of detectable ctDNA changes should be interpreted cautiously and integrated with radiographic and clinical information.

Foremost is the lack of definitional uniformity. The BR.36 trial defined molecular response as maximal mutant allele fraction clearance at cycle 3 day 1 (3), whereas the VHIO study operationalized molecular response as ≥30% decrease in ctDNA at pre-cycle 2 relative to baseline, molecular clearance as any undetectable timepoint, and durable molecular clearance as sustained undetectability for ≥180 days (4). In parallel, investigators have proposed alternative frameworks such as liquid biopsy–RECIST (LB-RECIST), which categorizes ctDNA changes into complete response, partial response, stable disease, and progression based on confidence interval overlap (6). This heterogeneity complicates cross-trial comparisons and meta-analytic validation. Beyond definitional heterogeneity, technical factors also influence the reliability of ctDNA-based monitoring. False-negative results may occur in tumors with limited ctDNA shedding, low metastatic burden, or anatomically confined disease such as isolated central nervous system metastases. Moreover, assay performance depends on multiple variables, including plasma input volume, sequencing depth, bioinformatic processing, and whether tumor-informed or tumor-agnostic strategies are applied. These technical considerations may contribute to discordance between molecular and radiographic assessments and highlight the necessity for standardized analytical frameworks. A systematic review and meta-analysis of ctDNA clearance as a predictor of pathologic complete response (pCR) in patients receiving neoadjuvant ICIs highlighted this challenge: although pooled sensitivity was high (0.98; 95% CI, 0.86–1.00), specificity was only 0.53 (95% CI, 0.37–0.69), with substantial inter-study heterogeneity (I² ≈ 70%) (7). The authors concluded that while the absence of ctDNA clearance reliably identifies patients unlikely to achieve pCR, ctDNA clearance itself lacks sufficient confirmatory power to guide treatment decisions independently. Additionally, the RECIST Working Group convened a consensus workshop in September 2022, subsequently publishing a comprehensive evaluation that acknowledged the potential of ctDNA to augment response measurements but emphasized unresolved clinical, technical, and statistical limitations, including assay sensitivity variations, tumor-informed versus tumor-agnostic approaches, and the need for prospective validation (8).

A second major limitation is the paucity of randomized evidence demonstrating that ctDNA-guided treatment decisions improve hard endpoints such as OS. To date, most data derive from single-arm phase 2 trials, retrospective cohorts, or correlative analysis embedded within larger studies. The PRELUCA trial (NCT05573189) represents a notable exception: this nationwide Danish randomized study is comparing ctDNA-guided versus standard-of-care monitoring in patients with advanced NSCLC receiving ICI monotherapy, with OS as the primary endpoint (9). Although the study design is methodologically rigorous, mature survival data are not yet available, and its findings will be pivotal in determining whether ctDNA-mR can serve as a decision-making tool rather than merely a prognostic biomarker. Similarly, in the adjuvant setting, the IMvigor011 trial demonstrated that ctDNA status could identify patients with muscle-invasive bladder cancer who derived enhanced benefit from atezolizumab (10), yet this represents patient selection rather than dynamic response adaptation. The FDA’s 2024 guidance on the use of ctDNA in early-stage solid tumor drug development encourages prospective incorporation of ctDNA endpoints but stops short of endorsing ctDNA-mR as a registrational surrogate (11). Future clinical validation should transition from observational association toward intervention-based evidence. Randomized trials comparing standard RECIST-guided management with ctDNA-integrated adaptive treatment strategies are essential to determine whether molecular monitoring can improve patient outcomes. Such trials should incorporate predefined ctDNA thresholds, standardized assay platforms, and clinically meaningful endpoints including progression-free survival, overall survival, treatment duration, quality of life, and toxicity reduction. Only through prospective intervention studies can ctDNA evolve from a prognostic biomarker into a clinically validated decision-support tool. In this context, we propose an interim framework—termed “ctDNA-enhanced RECIST”—in which ctDNA serves as a complementary adjunct: (i) for early efficacy screening at 3–6 weeks to identify potential nonresponders; (ii) as a decision aid in iuPD, where decreasing ctDNA supports treatment continuation and increasing ctDNA prompts confirmatory evaluation; and (iii) for treatment duration optimization, wherein sustained ctDNA negativity for ≥6 months may provide exploratory information for evaluating treatment duration strategies, pending prospective validation.

ctDNA molecular response represents a promising biomarker that provides complementary biological information beyond conventional anatomical imaging during immune checkpoint inhibitor therapy. Current prospective studies demonstrate consistent associations between early ctDNA dynamics, radiographic response, and survival outcomes; however, available evidence remains insufficient to establish ctDNA as a validated surrogate endpoint replacing RECIST.

The major barriers include heterogeneous response definitions, technical variability, tumor-specific shedding patterns, and the lack of randomized evidence demonstrating improved outcomes through ctDNA-guided treatment adaptation. Therefore, ctDNA should currently be integrated within a multimodal response assessment framework rather than considered a replacement for radiographic evaluation.

The proposed ctDNA-enhanced RECIST approach provides a pragmatic framework for incorporating molecular information into immunotherapy monitoring. Future randomized clinical trials will be essential to determine whether ctDNA-guided strategies can improve treatment selection, optimize treatment duration, and ultimately enhance patient outcomes.
